# Synergistic Lithium Alloying and Plating in 3D Cu/CNT/Sn Electrodes for Stable Lithium Metal Batteries

**DOI:** 10.1002/smll.202501292

**Published:** 2025-06-20

**Authors:** Sul Ki Park, Soochan Kim, Ruhan He, Kate Sanders, Uiseok Hwang, Zongfu An, Mahdi Hamidinejad, Joon‐wan Kim, Michael De Volder

**Affiliations:** ^1^ Department of Engineering University of Cambridge Cambridge CB3 0FS UK; ^2^ School of Semiconductor and Chemical Engineering Jeonbuk National University 567 Baekje‐daero, Deokjin‐gu Jeonju‐si 54896 Republic of Korea; ^3^ School of Chemical Engineering Sungkyunkwan University Suwon 16419 Republic of Korea; ^4^ Department of Mechanical Engineering University of Alberta 9211‐116 Street NW Edmonton Alberta T6G1H9 Canada; ^5^ Laboratory for Future Interdisciplinary Research of Science and Technology (FIRST) Institute of Integrated Research (IIR) Institute of Science Tokyo (SCIENCE TOKYO) J3‐12, 4259 Nagatsutacho, Midori‐ku Yokohama 226–8503 Japan; ^6^ The Faraday Institution Didcot OX11 0RA UK

**Keywords:** dual lithium storage, lithiophilic interface, lithium metal batteries, stable cycling, 3D structure

## Abstract

Anode‐less Li‐ion batteries, in which Li is reversibly plated onto and stripped from a metal current collector during charge and discharge, theoretically offer the highest possible anode energy density. However, such systems suffer from rapid self‐discharge, excessive solid electrolyte interphase (SEI) formation, and dendritic lithium growth, resulting in severe performance degradation and safety concerns. Here, these challenges are addressed by introducing a novel 3D current collector that enables energy storage via a hybrid mechanism of alloying and plating. The 3D current collectors are fabricated through two scalable electroplating processes involving a porous Cu plating process followed by a Sn surface coating, and are structurally reinforced with carbon nanotubes (CNTs) to form a mechanically robust and conductive scaffold. The relative contributions of the alloying and plating reactions to the cell capacity are modulated by adjusting the thickness of the Sn layer, which governs the extent of lithiation through alloy formation. By optimizing the capacity distribution between Sn alloying and Li plating, the resulting half‐cell exhibits stable cycling over 200 cycles with an average Coulombic efficiency of 93.9%, significantly outperforming a control cell with planar Cu foils, which retain only 71.3% efficiency after 110 cycles.

## Graphical abstract

A 3D Cu/CNT/Sn current collector enables synergistic Li alloying and plating in Li‐ion batteries. Tuning the Sn layer reduces  Li inventory loss, resulting in stable cycling for 200 cycles in Li half‐cells and 60 cycles in anode‐less cells, improving over stability and Coulombic efficiency observed with conventional Cu current collectors.

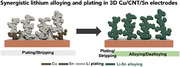

## 1 Introduction

The growing use of electric vehicles and new electronic devices imposes increasingly stringent demands on Li‐ion batteries (LiBs), driving the need for the development of higher‐energy‐density solutions. The vast majority of commercial LiBs are still relying on graphite anodes, similar to those commercialized over 30 years ago. While graphite achieves a decent capacity (LiC_6_, 372 mAh g^−1^),^[^ ^1^, ^2^, ^3^ ^]^ new anodes have been proposed with much improved performance. Among these, Li metal anodes are considered one of the most attractive anodes because of their low redox potential (−3.04 V vs. the standard hydrogen electrode) and ultrahigh theoretical specific capacity of 3860 mAh g^−1^.^[^ ^4^, ^5^ ^]^ Li metal anodes rely on Li plating and stripping to store and release charge during charging and discharging, respectively. Furthermore, all the Li for this process can be delivered by the cathode, meaning that the anode can be no more than a current collector, which could potentially lead to anode manufacturing savings. However, repeated Li plating and stripping result in the formation of excessive SEI, which consumes Li inventory, as well as in the growth of Li dendrites, which can lead to “dead Li” formation as well as short circuits.^[^ ^1^, ^3^, ^4^ ^]^

Various strategies have been introduced to address these challenges, such as new electrolyte formulations, artificial interfaces on the anode, and new structural designs of the anode.^[^ ^6^, ^7^, ^8^, ^9^, ^10^ ^]^ Among them, the new structural design of anodes, such as 3D nanostructures, is considered an efficient strategy for preventing the uncontrolled growth of Li.^[^ ^8^, ^11^, ^12^ ^]^ This strategy relies on the lower average current densities when using high surface area current collectors, leading to uniform dendrite‐free Li plating. As Cu is a standard anode current collector material, Cu‐based 3D electrodes have shown improvement in regulating the dendritic growth of Li metal; however, Cu is lithiophobic, which causes a high overpotential for Li metal nucleation.^[^ ^11^, ^12^, ^13^ ^]^ Therefore, it has been proposed to coat Cu current collectors with a lithiophilic metal coating to lower the Li nucleation over‐potential while maintaining the conductive metallic characteristics of the electrode.^[^ ^13^ ^]^ One particularly interesting approach is to use metal coatings that are both lithiophilic and allow for alloying reactions with Li. Such modification of the current collector allows to store Li both through alloying reaction and Li plating, so for the same stored capacity, the amount of Li plating is reduced, while at the same time the coating reduces the plating overpotential.^[^ ^14^, ^15^, ^16^ ^]^ Further, coated lithiophilic metal layers tend to swell substantially during alloying reactions, therefore they also profit from a thin 3D electrode structure to reduce mechanical stress and increase the cycling stability. In this paper, we leverage the synergies and similarities in requirements between alloying and plating reactions, and we balance the ratio between both processes to optimize the electrode cycling stability. Previous studies have primarily focused either on Li plating on 3D Cu frameworks to suppress dendrite growth^[^ ^17^, ^18^, ^19^, ^20^ ^]^ or on alloy‐based anodes such as Sn or Si to increase capacity and interfacial affinity with Li^[^ ^21^, ^22^, ^23^, ^24^ ^]^ However, these approaches tend to favor one reaction mechanism over the other, and few have investigated how the deliberate balance between Li alloying and plating within the same structure can improve electrochemical performance. Our work fills this critical gap by developing a rationally engineered 3D Cu/CNT/Sn framework, where the amount of Li that is stored via alloying (in Sn) and plating (on Cu) can be precisely controlled by adjusting the Sn layer thickness. This enables us to explicitly demonstrate how the synergistic interplay between the two mechanisms affects Coulombic efficiency (CE), cycle life, and structural stability.

In our approach, we fabricate 3D porous Cu/CNT composites using a scalable electroplating process reported in our previous research.^[^ ^8^ ^]^ The CNTs play a key role in improving the mechanical strength of the plated 3D Cu, and the Cu coating reduces the surface area of the CNTs. We then electroplate Sn (3D Cu/CNT/Sn) as a lithiophilic interphase that allows for alloying reactions. Among various metals such as Sn, Mg, Zn, Ag, Bi, etc., Sn is a promising anodic material for high‐performance Li metal batteries due to its high gravimetric capacity (Li_22_Sn_5_: 991 mAh g^−1^) and small interface impedance and fast Li‐ion diffusion.^[^ ^15^ ^]^ Moreover, Sn is known to exhibit high lithophilicity (−2.63 eV) and has been widely studied due to its relatively low cost compared to Ag, which shows similar lithophilicity (Ag: −2.17 eV, Zn: −1.93 eV).^[^ ^25^, ^26^, ^27^, ^28^ ^]^ In contrast, experimental observation (see Figure S1, Supporting Information) reveals poor Li plating behavior on Zn deposited Cu structure, further highlighting the practical advantages of Sn in achieving uniform Li deposition. Using a pulsed Sn electrodeposition, we coat different Sn thicknesses to investigate the influence of different ratios between Sn alloying and Li plating on the cycling stability. By varying the Sn composition, we systematically control the alloying‐to‐plating ratio and correlate this ratio with battery performance outcomes. The optimized 3D Cu/CNT/Sn electrode showed a CE of 93.9% during 200 cycles in half cells at a current density of 1 mA cm^−2^ (1 mAh cm^−2^) and an anode‐less Li metal full cell assembled with LiFePO_4_ (LFP) electrode also exhibits stable cycling behavior with 70.7% capacity retention after 60 cycles.

## 2 Results and Discussion

**Figure 1:**
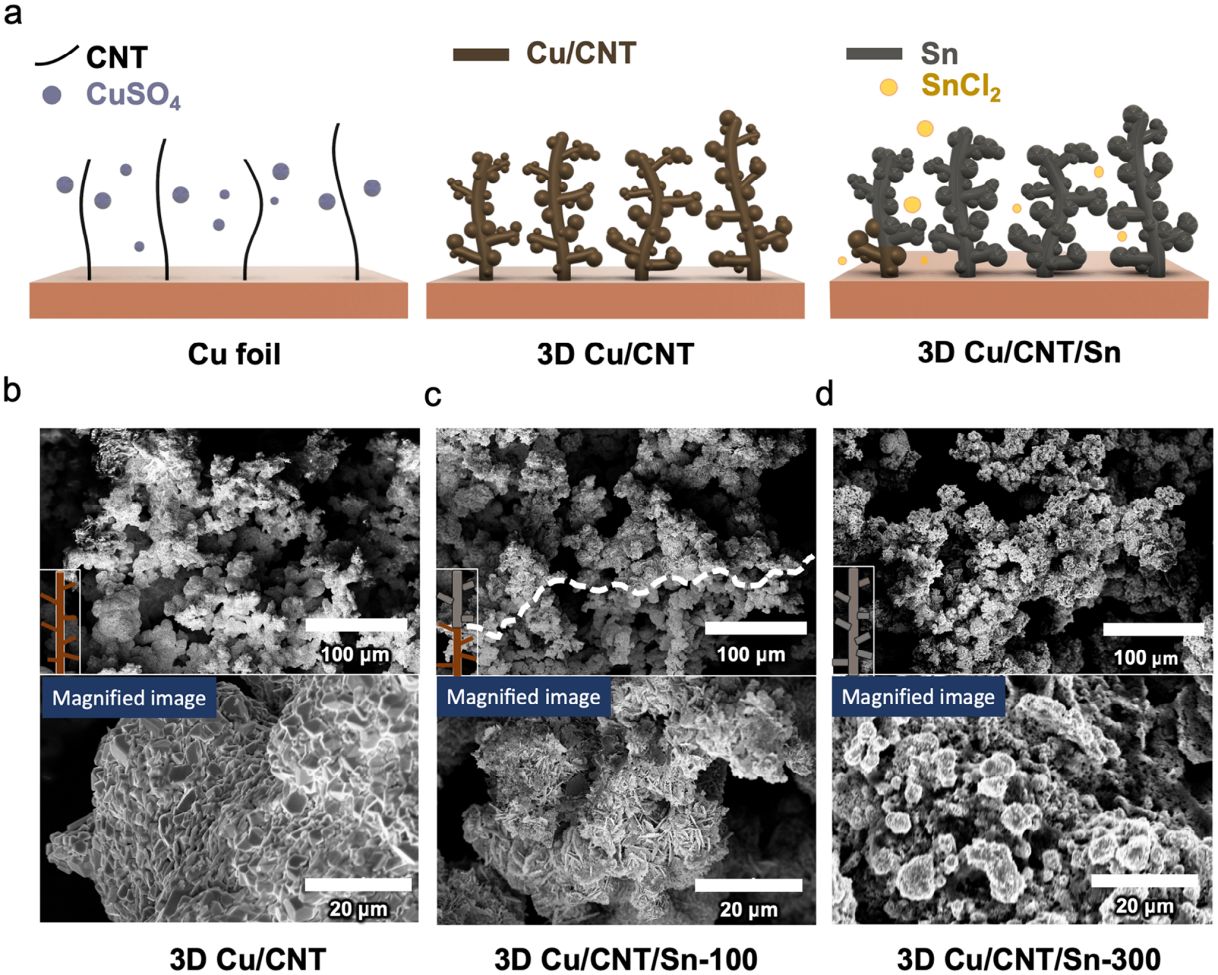
a) Schematic illustration of the fabrication process for 3D Cu/CNT/Sn composite electrodes, b–d) SEM images of different types of electrodes studied in this work with magnified images (3D Cu/CNT, 3D Cu/CNT/Sn‐100, and 3D Cu/CNT/Sn‐300, scale bar = 100 and 20 µm, respectively).

The 3D Cu/CNT/Sn electrodes are fabricated using a scalable electroplating process, as shown in **Figure** **1**a. A two‐electrode system was used for plating the 3D Cu/CNT structure, consisting of a Cu foil and a Cu plate counter electrode. The plating bath relies on an aqueous CuSO_4_ electrolyte in which oxidized multiwalled CNTs are dispersed. For the porous 3D Cu/CNT composites, a constant current set to 1.2 A cm^−2^ for 1100 s is used in agreement with our previous work.^[^ ^8^ ^]^ The Cu plates form a 3D porous matrix by virtue of the CNTs that are embedded in the Cu structure during plating, and form a branch along which the Cu plating can proceed. The embedding of CNTs in the Cu matrix provides a high surface area that would lead to excessive SEI formation. In addition to creating a 3D porous Cu structure, the CNTs increase the ductility of the plated material (Scanning Electron Microscope; SEM image and Energy‐Dispersive X‐ray Spectroscopy; EDS mapping in Figure S1a, Supporting Information).^[^ ^8^ ^]^ After the 3D Cu/CNT backbone is formed, the samples are dried and then transferred to a second electroplating station where they are coated with an Sn layer for energy storage through alloying reactions and to provide lithiophilic layers for Li plating. This coating is carried out using a pulsed electrodeposition process (5 mA cm^−2^ with 30s pulses and 30s rest periods). Pulse electrodeposition yields a finer‐grained and more homogenous Sn coating of the 3D Cu/CNT, and we will refer to these materials as 3D Cu/CNT/Sn.^[^ ^29^, ^30^ ^]^ As shown in Figure S2 (Supporting Information), a constant current Sn deposition for 150s at −1.8 V results in the agglomeration of Sn particles on the top of 3D Cu/CNT. Instead, pulsed Sn deposition proceeds evenly over the surface of the electrode which changes gradually from Cu to Sn (Figure 1b–d). During the Sn plating process, Sn was first deposited on top of the 3D Cu/CNT electrode and then proceeded downward. The 3D Cu/CNT/Sn‐100 structure (electrodeposition with 100 pulsed cycles) shows partial deposition of Sn particles on the top part of the electrode (Figure S3a–c, Supporting Information). The boundary between the part of the electrode coated by Sn is marked by a dotted line in Figure 1c and Figure S3b (Supporting Information). The electroplated Cu and Sn have a different morphology as illustrated in the high magnification inset images. After 300 pulses of electroplating (3D Cu/CNT/Sn‐300), Sn is deposited over the entire height of the electrode (Figure S3d–f, Supporting Information and EDS mapping in Figure S4, Supporting Information). However, an electrode with 500 electrodeposition cycles (3D Cu/CNT/Sn‐500) was fractured due to excessive Sn deposition (Figure S5, Supporting Information).

**Figure 2:**
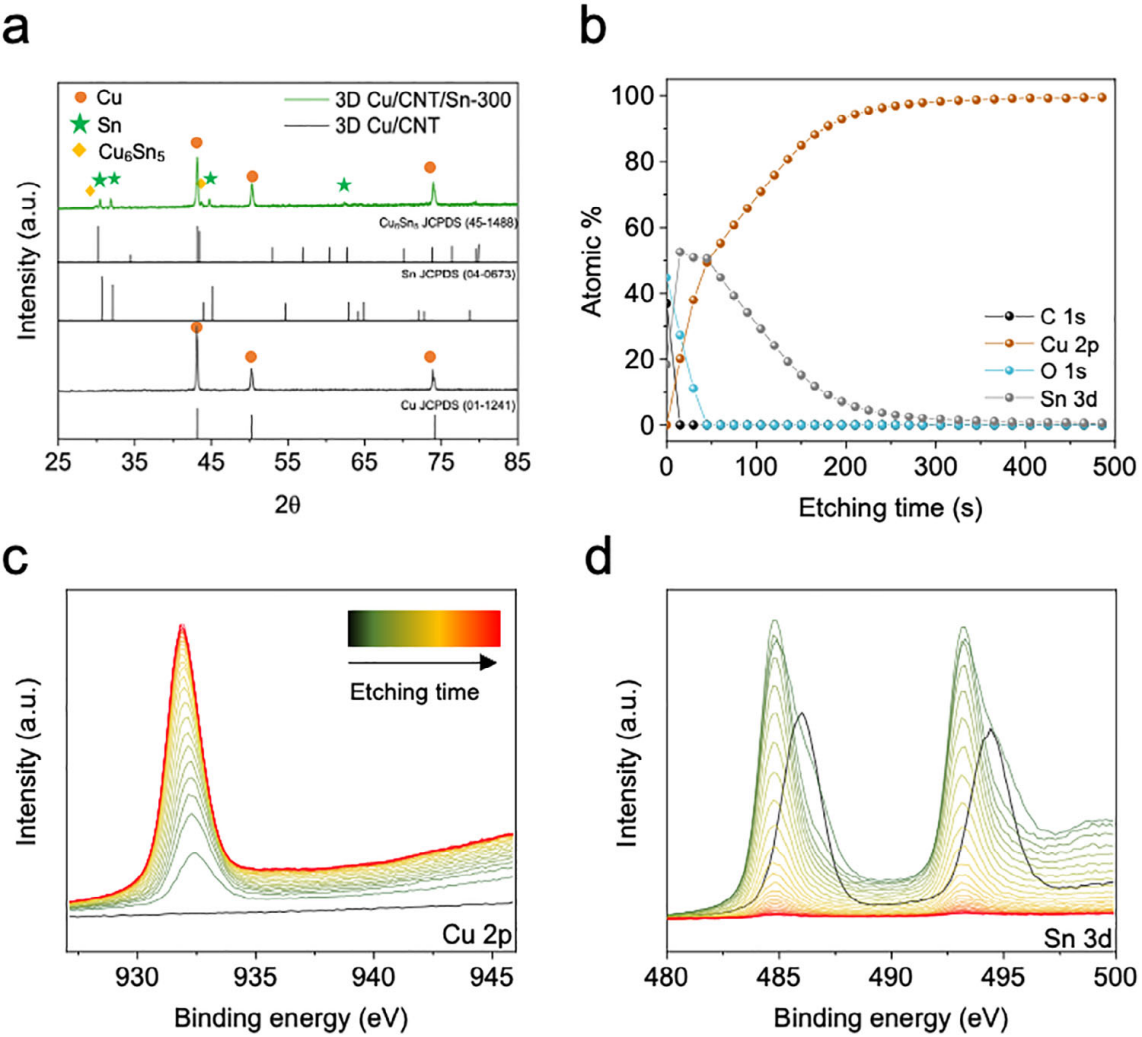
a) XRD patterns of 3D Cu/CNT and 3D Cu/CNT/Sn‐300 electrodes, b–d) XPS analysis for 3D Cu/CNT/Sn‐300. (b) Atomic ratios of Cu, Sn, and O as a function of Ar‐ion etching time and (c,d) Cu 2p and Sn 3d XPS profiles as a function of etching time with initial peak (black).

**Figures** **2**a and S6 (Supporting Information) show the X‐ray diffraction (XRD) patterns of the 3D Cu/CNT and 3D Cu/CNT/Sn electrodes, respectively. The diffraction peaks marked by orange, green, and yellow diamonds were assigned to face‐centered cubic Cu, tetragonal Sn, and monoclinic Cu_6_Sn_5_, respectively, according to the literature.^[^ ^31^, ^32^, ^33^ ^]^ Through this, we assume that a small amount of Cu─Sn alloy was formed at the interface between the Cu and Sn layers. To investigate the changes in the chemical composition of the 3D Cu/CNT/Sn electrode in detail, X‐ray photoelectron spectroscopy (XPS) depth profiling was conducted, as shown in Figure 2b–d. By tracking the Cu 2p, Sn 3d, and O 1s spectra at different etching times, the degree of Sn coating on the 3D Cu/CNT can be confirmed, and some layers can confirm the alloying of Cu─Sn. At the start of the etching process, metal oxide peaks were detected, probably due to oxidation of the samples when exposed to air. Once these oxide layers were etched away, the atomic ratio of Sn in 3D Cu/CNT/Sn‐300 decreased with increasing etching time, and the Cu atomic content increased, consistent with what is expected from the two‐step electroplating process.

**Figure 3:**
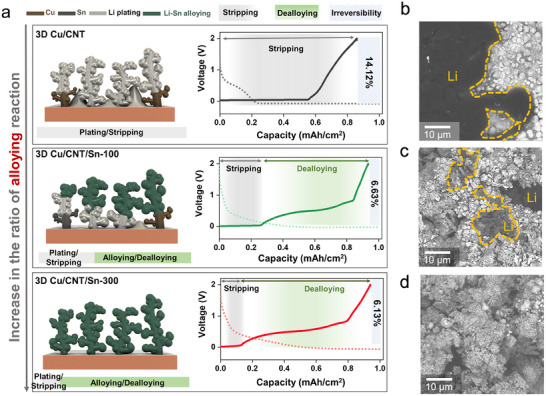
a) Schematic illustration of the lithiation process on our three electrode designs and b–d) SEM images of electrodes after Li metal plating at 1 mAh cm^−2^. (scale bar: 10 µm, 3D Cu/CNT, 3D Cu/CNT/Sn‐100, and 3D Cu/CNT/Sn‐300, respectively).

To investigate the energy storage process in our 3D Cu/CNT/Sn architectures, the different electrodes (3D Cu/CNT, 3D Cu/CNT/Sn‐100, and 3D Cu/CNT/Sn‐300) were lithiated/delithiated with a current density of 1 mAh cm^−2^ (**Figure** **3**a). During lithiation, the 3D Cu/CNT electrodes (Figure 3a) show a sharp voltage drop to below 0 V vs. Li/Li^+^, indicating that Li is almost immediately plated without alloying reactions taking place.^[^ ^34^ ^]^ Meanwhile, the 3D Cu/CNT/Sn voltage curves show both capacities stored above and below 0 V vs. Li/Li^+^, which reflects the combination of alloying reactions with Sn and Li plating, respectively. During de‐lithiation, the 3D Cu/CNT/Sn electrodes show dealloying and stripping processes with an increase in CE from 86% to 94% after Sn coating. Part of the poor CE in 3D Cu/CNT electrodes is caused by the non‐uniform lithiation/delithiation of Li metal, as shown in Figure 3b–d (SEM images after Li metal plating at 1 mAh cm^−2^). The top surfaces of the 3D Cu/CNT/Sn electrodes show improved reversibility of lithiation with increasing Sn deposition time, consistent with the assumption established earlier, indicating the synergistic effect between the Sn alloying process and Li metal plating on lithiophilic Sn layers. In addition, the lithiated 3D Cu/CNT/Sn electrode was characterized by XPS (Figure S7, Supporting Information). The XPS analysis reveals the presence of surface species such as Li₂O, LiF, LiOH, and Li─Sn alloy, which are indicative of SEI components likely originating from the reaction between Li and the electrolyte. Further, the peak shift in Sn 3d spectra was observed which it presented the formation of Li─Sn alloy.^[^ ^4^, ^35^, ^36^ ^]^

**Figure 4:**
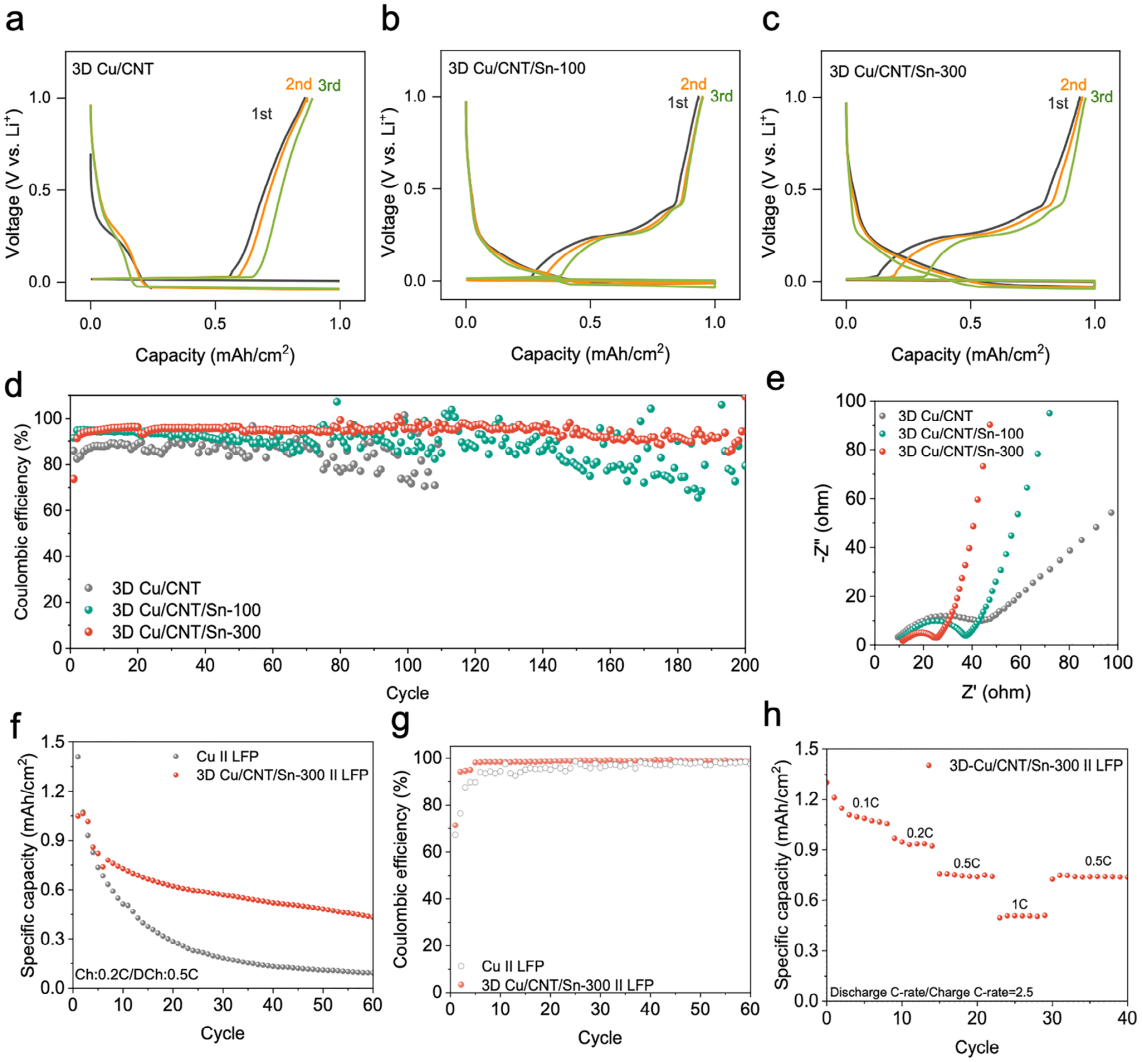
a–c) Galvanostatic charge‐discharge curves of the Li||3D Cu/CNT, Li||3D Cu/CNT/Sn‐100 and Li||3D Cu/CNT/Sn‐300 half‐cell at 1 mA cm^−2^ (1 mAh cm^−2^), d) CE of the Li||3D Cu/CNT, Li||3D Cu/CNT/Sn‐100, and Li||3D Cu/CNT/Sn‐300 half‐cells at 1 mA cm^−2^ (1 mAh cm^−2^), e) Nyquist plots of the half‐cells after 50 cycles, f) Cycling performance of assembled ALMB cells, g) CE of assembled ALMB cells, and h) Rate performance of 3D Cu/CNT/Sn‐300 || LFP cell.

To further investigate the cycling behavior of Li, we performed a cycling test of the half‐cells (Li||3D Cu/CNT, Li||3D Cu/CNT/Sn‐100, 300, 400, and 500) at 1 mA cm^−2^ and 1 mAh cm^−2^, as shown in **Figures** **4**a–d and S8 (Supporting Information). The Li||3D Cu/CNT half‐cell exhibited improved cycling behavior compared to the blank half‐cells (Li||Cu foil in Figure S9, Supporting Information), but short circuits of the cell occurred after 110 cycles. Meanwhile, the Li||3D Cu/CNT/Sn‐300 cells show stable cycling behavior over 200 cycles, probably owing to the spread of energy storage over alloying and plating processes and the lithiophilic properties of the Sn layer. The CE is a critical parameter for measuring the long‐term stable cycling of a working electrode. As shown in Figure 4d, the Li||3D Cu/CNT/Sn‐300 cell maintained a CE of 93.9% for up to 200 cycles, whereas the Li||3D Cu/CNT half‐cell only achieved a relatively low CE (70.3% after 100 cycles). Moreover, the cycled 3D Cu/CNT/Sn‐300 electrode (lithiated) maintained its structure without significant structural collapse (Figure S10, Supporting Information). Li||3D Cu/CNT/Sn‐400 and Li||3D Cu/CNT/Sn‐500 cells, which contain a higher Sn loading, exhibited increased cycling instability (Figure S8, Supporting Information). This behavior is attributed to structural degradation induced by excessive volume expansion of Sn during repeated lithiation/delithiation. In addition, Li||3D Cu/CNT/Sn‐300 cell also presented stable cycling behavior at high current density conditions (3 mA cm^−2^, 3 mAh cm^−2^), shown in Figure S11 (Supporting Information). These findings underscore the importance of optimizing Sn content to ensure structural integrity and stable cycling performance, considering cycling behaviors of various electrode designs with 3D metal/Cu structures (Table S1, Supporting Information).

Electrochemical Impedance Spectroscopy (EIS) is considered a nondestructive diagnostic tool to characterize battery electrodes within a short testing timeframe.^[^ ^37^, ^38^, ^39^ ^]^ In Figure 4e, after 50 cycles, Nyquist plots of half‐cells with Cu/CNT and Cu/CNT/Sn electrodes(100, 300) obtained by EIS show a lower resistance value than half cell with the 3D‐Cu/CNT electrode, which is affected by improved lithiophilic properties of the Sn layer (detailed in Figure S12, Supporting Information). Among them, the half‐cell with the optimized 3D‐Cu/CNT/Sn‐300 electrode exhibits the lowest resistance value, suggesting that this optimized electrode can provide an efficient electrochemical system when used as a stable Li metal anode. Therefore, these various electrochemical results indicate that the 3D Cu/CNT/Sn‐300 electrode can control the non‐uniform growth of Li, leading to long‐term cycling of Li metal‐based batteries. Inspired by the features of anodes based on dual lithiation system, a full‐cell has been assembled by employing a commercial LFP cathode (MTI Corp., single‐side‐coated electrode, and 12.9 mg cm^−2^ of LFP loading) with an advanced anode‐less Li metal battery (ALMB) system (Figure 4f–h). ALMBs utilize an anodic current collector for the plating and stripping of Li, eliminating the need for a conventional anode such as graphite, graphite/Si composite, etc. This innovative approach holds great promise for enhancing the energy density of batteries.^[^ ^4^ ^]^ The full cells were activated for 3 cycles and the cell with the 3D Cu/CNT/Sn‐300 showed improved cycling stability with a capacity retention of 70.7% after 60 cycles whereas a Cu foil reference only achieved 11.4% capacity retention at C/5 charge and C/2 discharge with the liquid electrolyte (1.3 M lithium hexafluorophosphate in a mixture of ethylene carbonate, ethyl methyl carbonate, and diethyl carbonate (3:5:2) in 0.2 wt.% lithium tetrafluoroborate, 10 wt.% fluoroethylene carbonate, and 0.5 wt.% vinylene carbonate). In addition, the rate performance of the ALMB cell with different separators was evaluated at various current densities (0.1 C, 0.2 C, 0.5 C, 1 C, and 0.5 C) in Figure 4h. The cell with the 3D Cu/CNT/Sn‐300 electrode exhibited high‐rate stability, without sudden short‐circuiting or significant capacity fading when the current density was returned to 0.5 C. The improved cycling stability of this full cell arises from the synergistic effect of our Cu/CNT/Sn system, and we anticipate that the cycling performance can be further improved by using new electrolyte formulations (e.g., localized high‐concentration electrolyte, dual‐salt‐based electrolyte), new battery structure, and tailored cycling protocols.^[^ ^4^, ^40^, ^41^, ^42^, ^43^, ^44^, ^45^ ^]^

## 3 Conclusion

In this study, we leverage the synergies in energy storage between Sn as a material for Li alloying and a 3D substrate for Li plating. Both these charge storage processes benefit from an increased surface area, for which we use a scalable electroplating process to create 3D Cu/CNT/Sn electrodes. The fabricated 3D Cu/CNT/Sn electrodes are mechanically stable thanks to the CNTs that are incorporated in the structure during the plating process. Our 3D Cu/CNT/Sn electrodes show stable cycling for over 200 cycles in half‐cells, with an average Coulombic efficiency of 93.9%, whereas reference experiments with a Cu foil achieve only 71.3% after 110 cycles. In ALMBs versus an LFP cathode, we achieve improved stability in the battery cycling for 60 cycles compared to the full cell with the reference Cu electrode. These findings show the merits of combining different storage mechanisms in combination with 3D electrode architectures.

## 4 Experimental Section

### Fabrication of 3D Cu/CNT

A 3D Cu/CNT electrode was fabricated via electrodeposition considering the previous work.^[^ ^8^ ^]^ A two‐electrode setup was used in which the working electrode was a clean Cu foil and the counter electrode was a Cu plate. The electrolyte was a 0.5 m copper sulfate solution in sulfuric acid and a 0.1 wt.% CNT dispersion (2 mL dissolved in DI water. The 3D porous Cu/CNTs were prepared by electrodeposition at 1.2 A cm^−2^ for 1000 s in the electrolyte. The as‐synthesized 3D Cu/CNT electrode was dried at 60 ^°^C overnight.

### Fabrication of 3D Cu/CNT/Sn Electrodes

3D Cu/CNT/Sn electrodes were synthesized via electrodeposition. A three‐electrode setup was used, in which the working electrode was the as‐prepared 3D Cu/CNT, the reference electrode was Ag/AgCl, and the counter electrode was platinum (Pt) wire. The electrolyte was a 0.01 m SnCl_2_ solution in DI water. The 3D Cu/CNT/Sn was prepared by electrodeposition at 5 mA cm^−2^ for 30s on and off for 100, 300, 400, and 500 cycles, naming these 3D Cu/CNT/Sn−100, −300, −400, and −500 respectively.

### Electrochemical Characterizations

CR 2032‐coin cells were assembled for the electrochemical measurements in an Ar‐filled glove box. Microporous polyolefin (Celgard 2325) was used as the separator. The electrolyte was 1.3 m lithium hexafluorophosphate (LiPF_6_) in a mixture of ethylene carbonate (EC), ethyl methyl carbonate (EMC), and diethyl carbonate (DEC) (3:5:2) in 0.2 wt.% LiBF_4_, 10 wt.% fluoroethylene carbonate (FEC), and 0.5 wt.% Vinylene carbonate (VC) was used as a liquid electrolyte. The electrochemical properties of the half‐cell of 3D Cu/Sn were investigated under the lithiation capacity cut‐off up to 0.1 mAh cm^−2^ with a current density of 0.1 mA cm^−2^ and delithiation up to 2.0 V with 0.005 mA cm^−2^ for three formation cycles and then cycled at a current density 0.05 mA cm^−2^. The cycle stabilities of 3D Cu/CNT and 3D Cu/CNT/Sn were conducted during charging under the capacity cut‐off up to 1 mAh cm^−2^ (3 mAh cm^−2^) with a current density of 1 mA cm^−2^ (3 mA cm^−2^) and discharging up to 2.0 V. For assembly of ALMBs, commercial lithium iron phosphate (LiFePO_4_, LFP) cathode (MTI corp., single‐side coated electrode, and 12.9 mg cm^−2^ of active material loading) and as‐fabricated 3D Cu/CNT/Sn‐300 anode were assembled in CR 2032‐coin cells with 50 µL of the electrolyte. The full cells were tested with the asymmetric cycling protocol (charged to 3.8 V at C/5 and discharged to 2.5 V at C/2). Cell testing was performed via galvanostatic charge‐discharge on a BCS 805 series battery cycler (Biologic, France), and the cells were placed in an environmental chamber at 26 °C. A VMP3 potentiostat (BioLogic, France) was used for electrochemical impedance spectroscopy (EIS), which was conducted by applying a 10‐mV amplitude sine wave in the frequency range (10 mHz–10 kHz).

### Material Characterizations

The morphologies of the prepared electrodes were observed using scanning electron microscopy (SEM, Phenom Pro SEM, Thermo Fisher, USA, at 10.0 kV in backscattering mode. To prepare Li metal‐containing samples for SEM, the cells were disassembled in an argon‐filled glove box, rinsed with dimethyl carbonate (DMC, anhydrous, Sigma–Aldrich, USA) to remove residual salt, and dried. Energy dispersive X‐ray spectroscopy (EDS) was measured by SUPRA40VP (Zeiss, Germany). X‐ray photoelectron spectroscopy (XPS) depth profiling with ion etching was performed using a NEXSA G2 surface analysis system (Thermo Fisher Scientific, USA). The system utilized monochromatized Al Kα X‐rays with an energy of 1486.7 eV, allowing for precise measurements of core levels including Cu 2p, Sn 3d, Li 1s, F 1s, and O 1s. To ensure rapid data acquisition, each core‐level spectrum was conducted in snap mode, leveraging a 128‐channel detector with a dwell time of 20s per spectrum. The analysis chamber was maintained at a stable base pressure of 10^−9^ mbar, and all measurements took place at room temperature (25 °C). For air‐sensitive samples, vacuum transfer module (Thermo Fisher Scientific, USA) was used. X‐ray diffraction analysis (XRD, Smartlab, Rigaku, Japan) was conducted with Cu Kα radiation. Patterns were collected from 20° to 80° at a scan rate of 5° min^−1^.

## Conflict of Interest

The authors declare no conflict of interest.

## Supporting information



Supporting Information

## Data Availability

The data that support the findings of this study are available from the corresponding author upon reasonable request.
