# Supplementary material for: Synergistic Lithium Alloying and Plating in 3D Cu/CNT/Sn Electrodes for Stable Lithium Metal Batteries
Source: Small. 2025 Jun 20;21(33):2501292. doi: 10.1002/smll.202501292 (PMC12372435; doi:10.1002/smll.202501292)
Supplement: Supplementary file 1 — Supporting Information [file SMLL-21-2501292-s001.docx]

**Supporting Information**

**Synergistic lithium alloying and plating in 3D Cu/CNT/Sn electrodes for stable lithium metal batteries**

Sul Ki Park, Soochan Kim, Ruhan He, Kate Sanders, Uiseok Hwang, Zongfu An, Mahdi Hamidinejad, Joon-wan Kim, Michael De Volder^*^

Dr. S. K. Park, Dr. R. He, Dr. K. Sanders, Prof. M. D. Volder

Department of Engineering, University of Cambridge, Cambridge CB3 0FS, United Kingdom E-mail: [mfld2@cam.ac.uk](mailto:mfld2@cam.ac.uk) (Prof. M. D. Volder)

Dr. S. K. Park

School of Chemical Engineering, Jeonbuk National University, 567 Baekje-daero, Deokjin-gu, Jeonju-si, Jeollabuk-do, 54896, Republic of Korea

Dr. U. Hwang, Dr. Z. An, Prof. S. Kim

School of Chemical Engineering, Sungkyunkwan University, Suwon 16419, Republic of Korea

Prof. M. Hamidinejad

Department of Mechanical Engineering, University of Alberta, 9211-116 Street NW, Edmonton, Alberta T6G1H9, Canada

Prof. J. Kim

Laboratory for Future Interdisciplinary Research of Science and Technology (FIRST), Institute of Integrated Research (IIR), Institute of Science Tokyo (SCIENCE TOKYO), J3-12, 4259 Nagatsutacho, Midori-ku, Yokohama, 226–8503 Japan

Prof. M. D. Volder

The Faraday Institution, Didcot OX11 0RA, United Kingdom

**1. Figures**


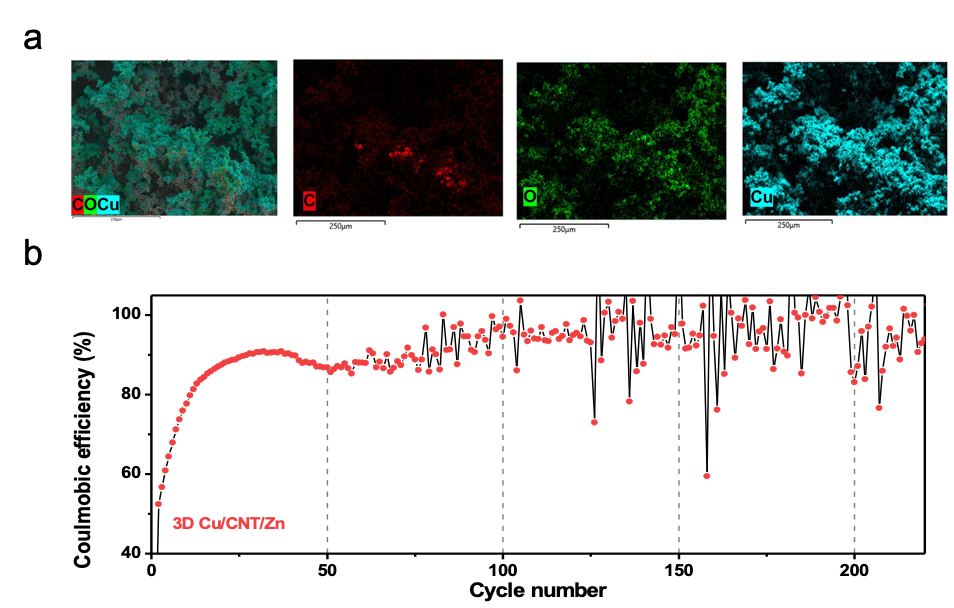


**Figure S1**. (a) EDS mapping of the 3D Cu/CNT electrode. [1] (Copyright 2023 American Chemical Society) and (b) Coulombic efficiency (CE) of the Li||3D Cu/CNT/Zn half-cell at 1 mA/cm^2^ (1 mAh/cm^2^)

**
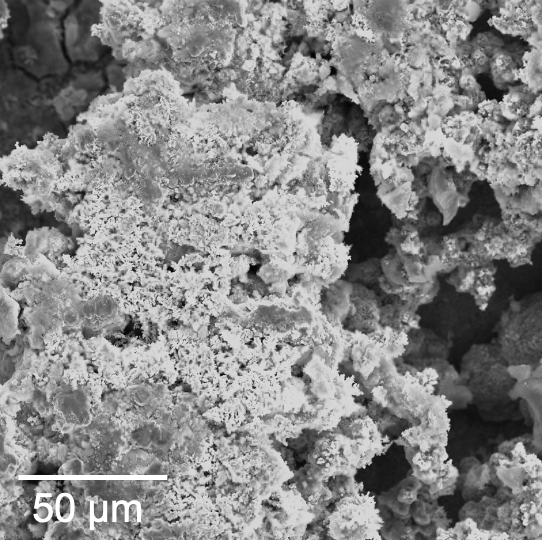
**

**Figure S2.** SEM image of continuous current deposited Sn onto 3D Cu electrode for 150s at 1.8V (scale bar: 50 μm)

**
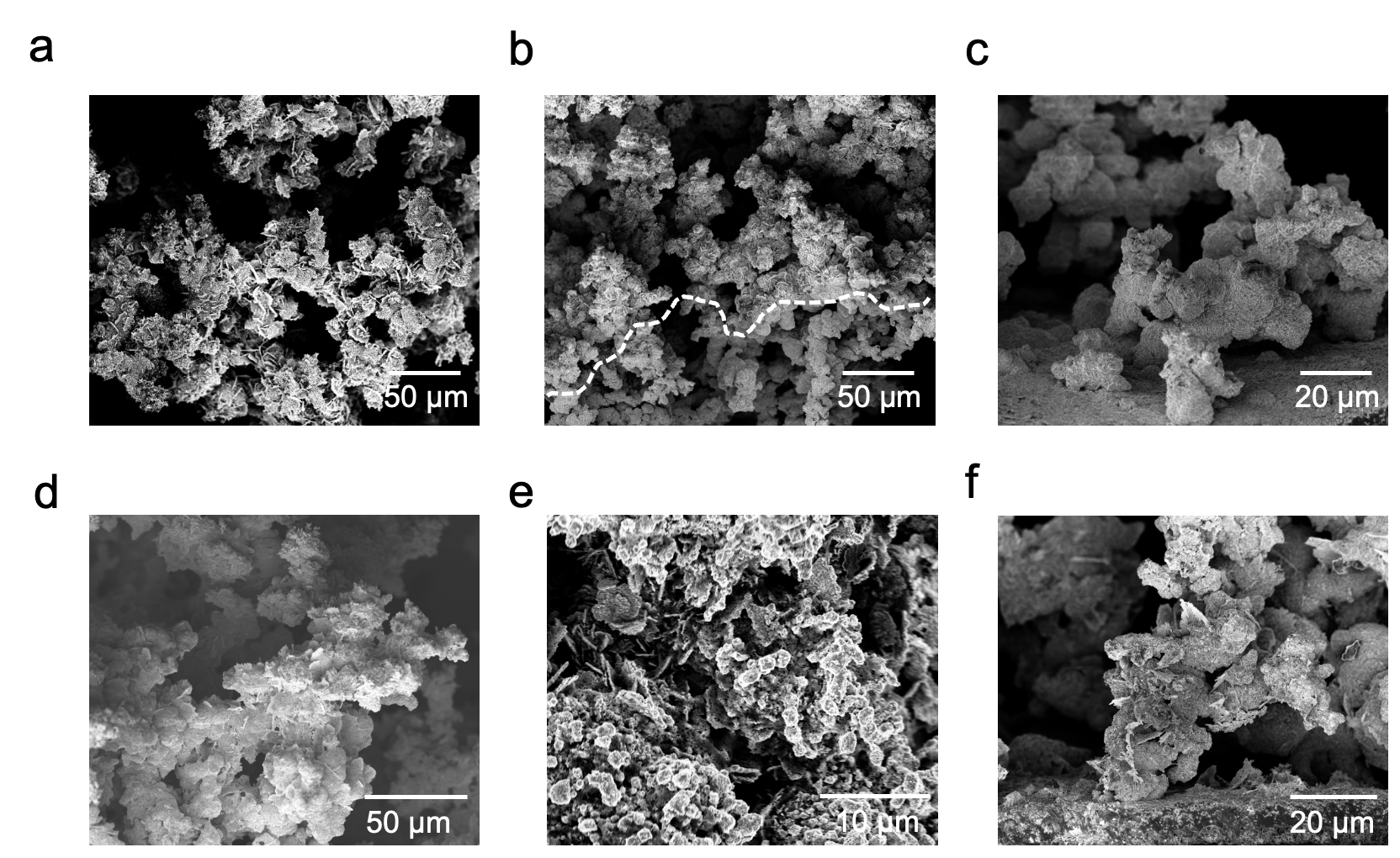
**

**Figure S3.** SEM images of 3D Cu/CNT/Sn electrodes. (a-c) 3D Cu/CNT/Sn-100 (top, middle, and bottom region, respectively) and (d-f) 3D Cu/CNT/Sn-300 (top, middle, and bottom region, respectively).


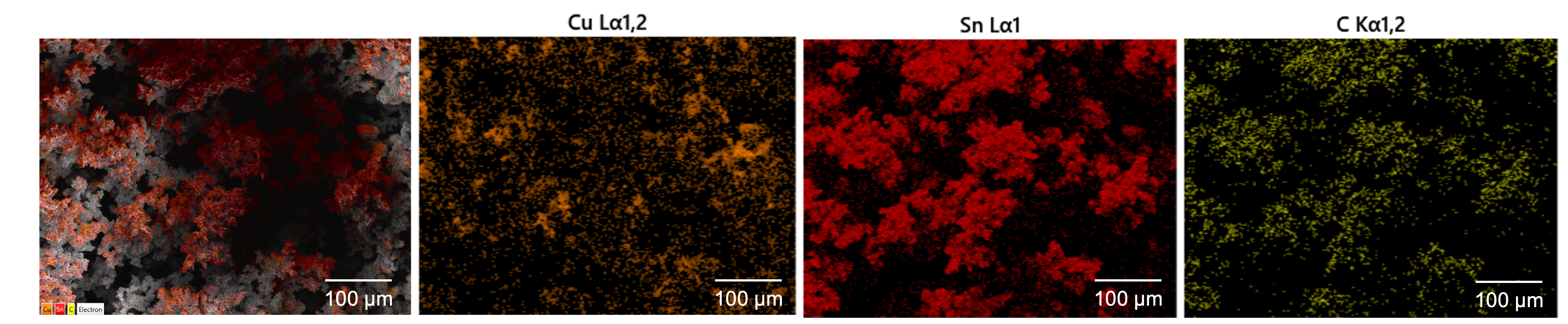


**Figure S4**. EDS mapping of the 3D Cu/CNT/Sn-300 electrode (scale bar=100 µm)

**
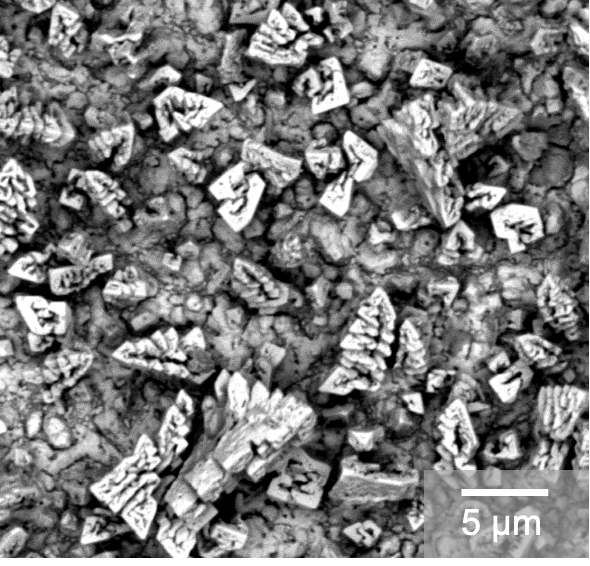
**

**Figure S5.** SEM image of the 3D Cu/CNT/Sn-500 electrode.


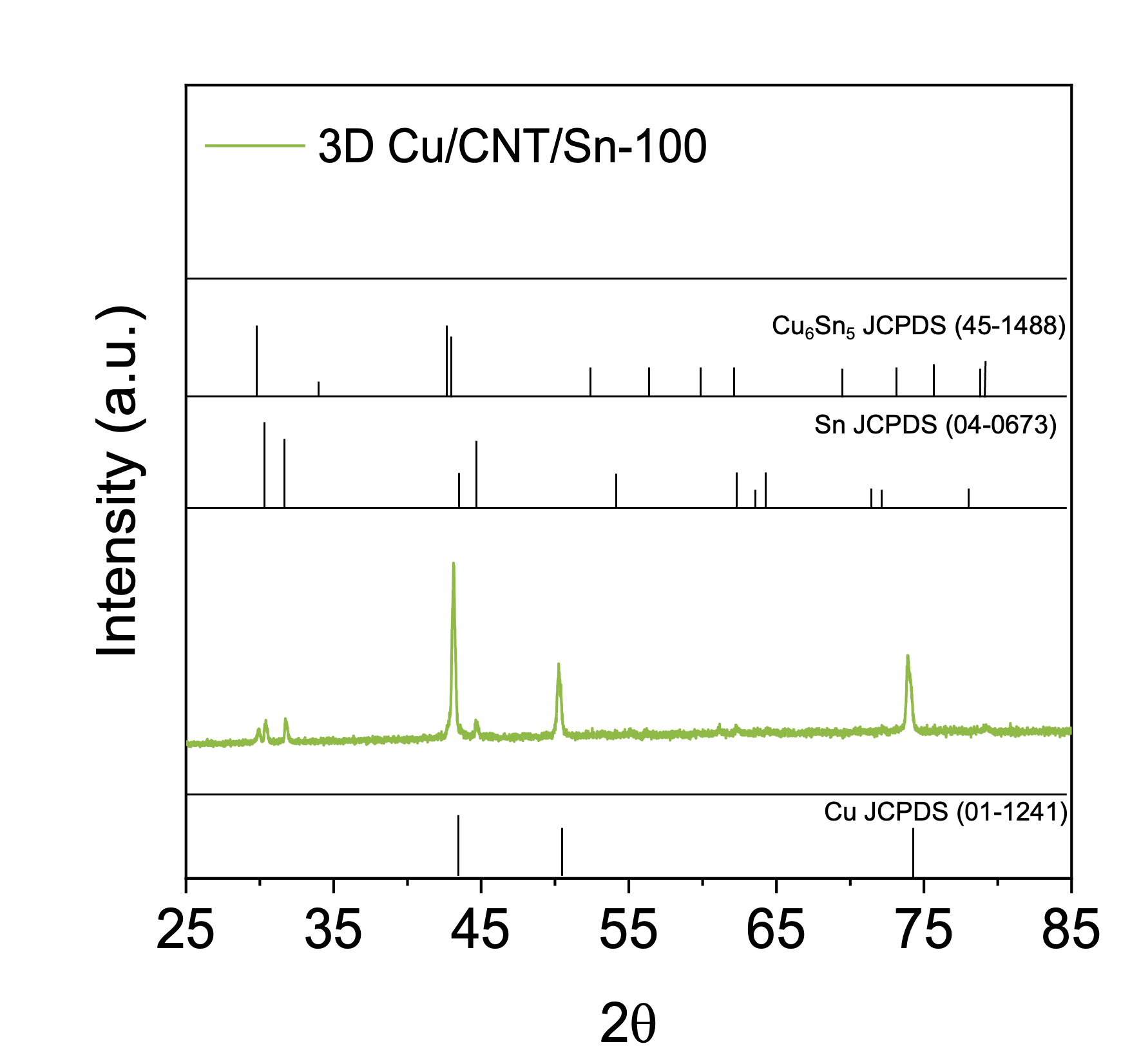


**Figure S6**. XRD pattern of the 3D Cu/CNT/Sn-100 electrode.


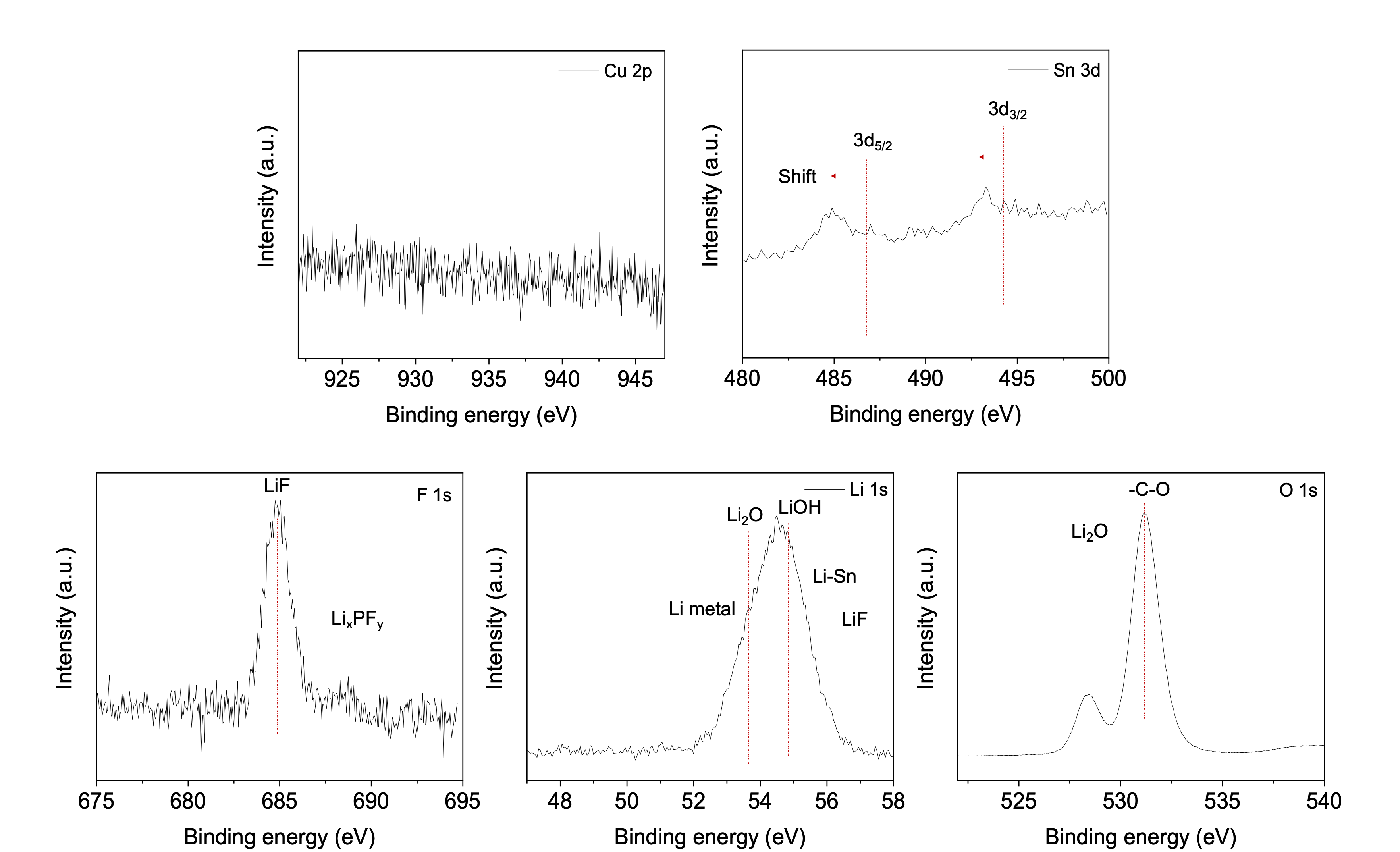


**Figure S7.** XPS analysis of the 3D Cu/CNT/Sn-300 electrode after the lithiation (Cu 2p, Sn 3d, F 1s, Li 1s, and O 1s)

**Figure S8**. Coulombic efficiency of half cells (Li||Cu/CNT/Sn-400 and Li||Cu/CNT/Sn-500) at 1 mA/cm^2^ (1 mAh/cm^2^)

**Figure S9**. Coulombic efficiency of the Li||blank Cu foil half-cell at 1 mA/cm^2^ (1 mAh/cm^2^)


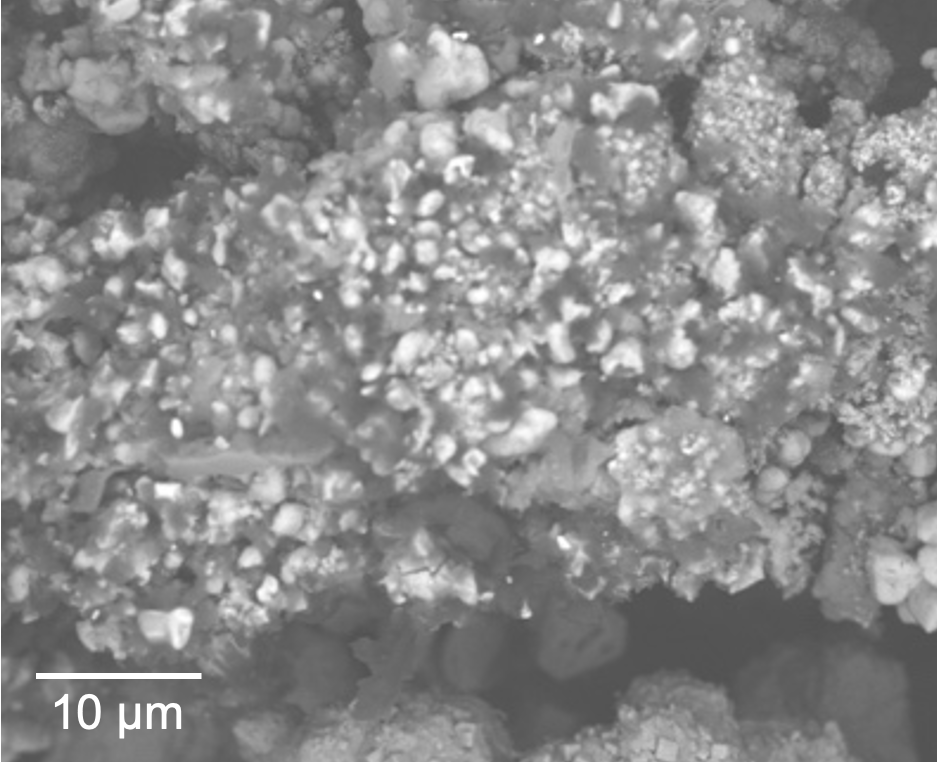


**Figure S10**. SEM image of cycled 3D Cu/CNT/Sn-300 electrode (after 200 cycles, lithiated)

**Figure S11**. Coulombic efficiency of the Li||Cu/CNT/Sn-300 half-cell at 3 mA/cm^2^ (3 mAh/cm^2^)


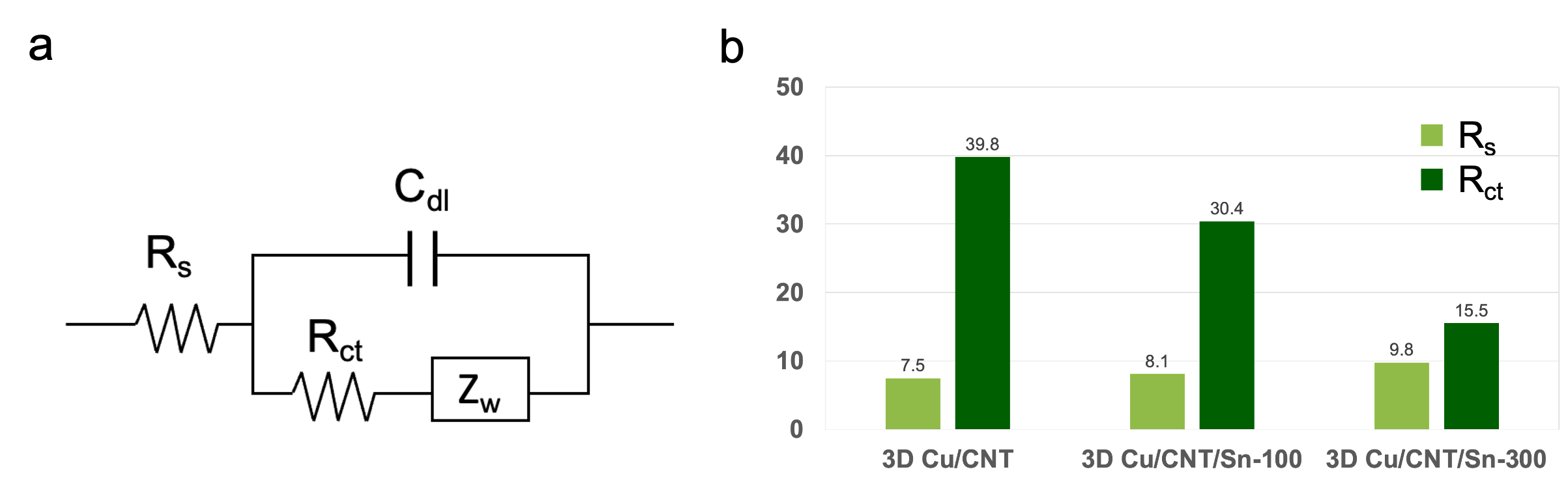


**Figure S12.** (a) EIS equivalent circuit to analyze obtained Nyquist plots in Figure 4e and (b) detailed equivalent circuit parameter (R_s_ and R_ct_)

The Nyquist plot was fitted by the equivalent circuit (Figure S12a), called Randles circuit model. This model is usually represented by R_s_ (electrolyte resistance), R_ct_ (charge-transfer resistance), C_dl_ (double layer capacitor), and Z_w_ (warburg constant). [2] Among these parameters, we focused on R_s_ and R_ct_, which related to the characteristics on the electrode interface. [2] In the case of the 3D Cu/CNT electrode, the charge transfer resistance was the highest, indicating that the large interfacial resistance may lead to an unfavorable structure for lithium-ion deposition. In contrast, the 3D Cu/CNT/Sn-300 electrode exhibited a lower interfacial resistance, suggesting that it enables stable Li deposition.

**Table S1.** Half-cell cycling performance (Li||metal coated porous Cu electrode) in recent published literature

| **Electrode** | **Current density** | **Number of cycles** | **Coulombic efficiency (%)** | **Reference** |
| --- | --- | --- | --- | --- |
| Ag-modified Cu foam | 0.5 mA/cm^2^  (2 mAh/ cm^2^) | 70 | Not mentioned | [3] |
| 3D porous CuZn | 1 mA/ cm^2^  (1 mAh/ cm^2^) | 150 | 95% after 150 cycles | [4] |
| 3D Cu@Sn | 0.5 mA/ cm^2^  (2 mAh/ cm^2^) | 160 | 98% after 160 cycles | [5] |
| 1D@3D-Cu/Sb | 1 mA/ cm^2^  (1 mAh/ cm^2^) | 140 | 96.8 % after 140 cycles | [6] |
| Nickel Inverse opal on Cu | 1 mA/ cm^2^  (1 mAh/ cm^2^) | 125 | 80% after 125 cycles | [7] |
| Au on copper mesh | 0.5C | 50 | 92.9 after 50 cycle | [8] |
| 3D Cu/CNT/Sn-300 | 1 mA/ cm^2^  (1 mAh/ cm^2^) | 200 | 93.9% after 200 cycles | Our work |

**Reference**

(1) Park, S. K.; Copic, D.; Zhao, T. Z.; Rutkowska, A.; Wen, B.; Sanders, K.; He, R.; Kim, H.-K.; De Volder, M. 3D Porous Cu-Composites for Stable Li-Metal Battery Anodes. *ACS Nano* **2023**, *17* (15), 14658-14666. DOI: 10.1021/acsnano.3c02223.

(2) Lazanas, A. C.; Prodromidis, M. I. Electrochemical Impedance Spectroscopy─A Tutorial. *ACS Measurement Science Au* **2023**, *3* (3), 162-193. DOI: 10.1021/acsmeasuresciau.2c00070.

(3) Zhu, Z.-W.; Wang, Z.-Y.; Liu, S.; Li, G.-R.; Gao, X.-P. Uniform lithium plating within 3D Cu foam enabled by Ag nanoparticles. *Electrochimica Acta* **2021**, *379*, 138152. DOI: https://doi.org/10.1016/j.electacta.2021.138152.

(4) Zhang, D.; Dai, A.; Wu, M.; Shen, K.; Xiao, T.; Hou, G.; Lu, J.; Tang, Y. Lithiophilic 3D Porous CuZn Current Collector for Stable Lithium Metal Batteries. *ACS Energy Letters* **2020**, *5* (1), 180-186. DOI: 10.1021/acsenergylett.9b01987.

(5) Guan, R.; Liu, S.; Wang, C.; Yang, Y.; Lu, D.; Bian, X. Lithiophilic Sn sites on 3D Cu current collector induced uniform lithium plating/stripping. *Chemical Engineering Journal* **2021**, *425*, 130177. DOI: https://doi.org/10.1016/j.cej.2021.130177.

(6) Fu, X.; Shang, C.; Zhou, G.; Wang, X. Lithiophilic Sb surface modified Cu nanowires grown on Cu foam: a synergistic 1D@3D hierarchical structure for stable lithium metal anodes. *Journal of Materials Chemistry A* **2021**, *9* (44), 24963-24970, 10.1039/D1TA08004K. DOI: 10.1039/D1TA08004K.

(7) Jeong, S. M.; Wu, M.; Kim, T. Y.; Kim, D. H.; Kim, S.-H.; Choi, H. K.; Kang, Y. C.; Kim, D. Y. A 3D Porous Inverse Opal Ni Structure on a Cu Current Collector for Stable Lithium-Metal Batteries. *Batteries & Supercaps* **2022**, *5* (3), e202100257. DOI: https://doi.org/10.1002/batt.202100257.

(8) Kim, E.; An, M.; Lee, C.; An, H.; Son, Y. Physically Coated Gold Film on Copper Mesh Electrode for Anode-Free Batteries. *ACS Applied Energy Materials* **2025**. DOI: 10.1021/acsaem.5c00923.
